# In Silico and In Vitro Studies of Mycotoxins and Their Cocktails; Their Toxicity and Its Mitigation by Silibinin Pre-Treatment

**DOI:** 10.3390/toxins12030148

**Published:** 2020-02-28

**Authors:** Van Nguyen Tran, Jitka Viktorova, Katerina Augustynkova, Nikola Jelenova, Simona Dobiasova, Katerina Rehorova, Marie Fenclova, Milena Stranska-Zachariasova, Libor Vitek, Jana Hajslova, Tomas Ruml

**Affiliations:** 1 Department of Biochemistry and Microbiology, University of Chemistry and Technology, Technicka 3, 16628 Prague 6, Czech Republic; tranv@vscht.cz (V.N.T.); prokesoj@vscht.cz (J.V.); katerina.augustynkova@gmail.com (K.A.); jelenovn@vscht.cz (N.J.); dobiasos@vscht.cz (S.D.); rehorova@vscht.cz (K.R.); 2 Department of Food Analysis and Nutrition, University of Chemistry and Technology, Technicka 3, 16628 Prague 6, Czech Republic; fenclovm@vscht.cz (M.F.); zacharim@vscht.cz (M.S.-Z.); hajslovj@vscht.cz (J.H.); 3 First Faculty of Medicine, Charles University, Katerinska 32, 12108 Prague 2, Czech Republic; vitek@cesnet.cz; 4 Faculty General Hospital, U Nemocnice 2, 12808 Praha 2, Czech Republic

**Keywords:** acute toxicity, combined toxicity, genotoxicity, cell protection, silibinin, in silico prediction, co-culture models

## Abstract

Mycotoxins found in randomly selected commercial milk thistle dietary supplement were evaluated for their toxicity in silico and in vitro. Using in silico methods, the basic physicochemical, pharmacological, and toxicological properties of the mycotoxins were predicted using ACD/Percepta. The in vitro cytotoxicity of individual mycotoxins was determined in mouse macrophage (RAW 264.7), human hepatoblastoma (HepG2), and human embryonic kidney (HEK 293T) cells. In addition, we studied the bioavailability potential of mycotoxins and silibinin utilizing an in vitro transwell system with differentiated human colon adenocarcinoma cells (Caco-2) simulating mycotoxin transfer through the intestinal epithelial barrier. The IC_50_ values for individual mycotoxins in studied cells were in the biologically relevant ranges as follows: 3.57–13.37 nM (T-2 toxin), 5.07–47.44 nM (HT-2 toxin), 3.66–17.74 nM (diacetoxyscirpenol). Furthermore, no acute toxicity was obtained for deoxynivalenol, beauvericin, zearalenone, enniatinENN-A, enniatin-A1, enniatin-B, enniatin-B1, alternariol, alternariol-9-methyl ether, tentoxin, and mycophenolic acid up to the 50 nM concentration. The acute toxicity of these mycotoxins in binary combinations exhibited antagonistic effects in the combinations of T-2 with DON, ENN-A1, or ENN-B, while the rest showed synergistic or additive effects. Silibinin had a significant protective effect against both the cytotoxicity of three mycotoxins (T-2 toxin, HT-2 toxin, DAS) and genotoxicity of AME, AOH, DON, and ENNs on HEK 293T. The bioavailability results confirmed that AME, DAS, ENN-B, TEN, T-2, and silibinin are transported through the epithelial cell layer and further metabolized. The bioavailability of silibinin is very similar to mycotoxins poor penetration.

## 1. Introduction

Mycotoxins, toxic secondary metabolites produced by fungi, are contaminants that frequently occur in food and feed worldwide. The mycotoxigenic fungal genera involved in the human food chain are mainly *Fusarium*, *Aspergillus*, *Penicillium*, and *Alternaria* [1]. Trichothecenes and zearalenone (ZEA) belong to the most important classes of mycotoxins produced by *Fusarium* species [2]. Depending on their functional groups, trichothecenes have been divided into Groups A–D [3]. T-2 toxin (T-2), HT-2 toxin (HT-2), and diacetoxyscirpenol (DAS) are the main representatives of the Type A subgroup [4,5]. Deoxynivalenol (DON), also known as vomitoxin, is the most prevalent mycotoxin of the Type B trichothecenes [6]. Besides, Fusarium also produces emerging fusariotoxins such as beauvericin (BEA) and enniatins (ENNs) [7]. BEA and ENNs are cyclic depsipeptides, which consist of free electron pairs of oxygen carbonyl groups and tertiary amino groups of amide bonds giving these molecules the ability to act as nucleophiles [8]. Alternaria fungi contaminate a wide variety of food items such as cereals, fruits, wheat, barley, and sorghum, where it produces several toxins, with alternariol (AOH), alternariol-9-methyl ether (AME), and tentoxin (TEN) being the most important ones [9]. Penicillium species are known to produce mycophenolic acid (MPA) [10]. Despite their low acute cytotoxicity on human cell line compared to other mycotoxins, MPA has been shown to possess neurotoxic and immunosuppressive effects [11]. The effects of selected mycotoxins on cell functions are listed in Table 1.

Although the main targets of mycotoxins are different, some of them have similar modes of actions and thus some additive effects of certain mycotoxins may be expected. Therefore, the presence of mycotoxins in plant products contaminated by several toxigenic fungi is an increasing health issue. Numerous studies have shown potential additive and even synergistic toxic effects of mycotoxins in vitro, summarized, e.g., in [32]. However, data on combined toxic effects of mycotoxins are generally limited and inconsistent. Most available publications in this field are dedicated to trichothecenes [33,34]. Moreover, reported studies focusing on the combined toxic effects of mycotoxins are incomparable to some extent due to the different experimental designs and conditions. For instance, the mixture of ZEA and DON showed a synergistic toxic effect in human hepatoblastoma HepG2 and RAW macrophage cells [18] but antagonistic effect in Bluegill fin fibroblast (BF-2 cells) [33]. Another study shows that, after 72 h of exposure, the combination of DON and T-2 toxins presented antagonistic effects in mammalian kidney epithelial (Vero) and Chinese hamster ovarian (CHO-K1) cells [14,23], while additive effects were observed in HepG2 cells [1]. In addition, the interaction between DON and T-2 varied from antagonism to synergism depending on the concentration and the ratio of mixtures. In general, in vitro studies of mycotoxin interactions have been mainly performed on single target cell lines. However, this does not fully mimic human metabolism or the complex interactions within the whole organism. Among other effects, such studies neglect intestinal epithelial transport and the first-pass hepatic metabolism [35].

Recently, the protective effects of silymarin or silibinin against mycotoxins have been reported in several publications. Silymarin extracted from seeds of *Silybum marianum* contains silibinin, isosilibinin, silydianin, and silychristin [36]. Silibinin, a major pharmacologically active compound, is a mixture of silybin A and silybin B. The studies of hepatoprotective effect of silymarin against fumonisin B1 (FB1) and aflatoxin B1 (AFB1) were performed in mice and bovine calves [37,38]. The FB1-induced hepatocyte damage was significantly diminished by the silymarin treatment. Silymarin decreased apoptosis rate, increased cell proliferation, and prevented the FB1-induced increase of TNF-α [37,39]. According to Naseer et al. [38], silymarin showed better results compared to choline chloride (liver tonics) in lowering the AFB1-induced serum aminotransferase, creatinine, and blood urea nitrogen. Silibinin has received much attention, but the negation of mycotoxins toxicity by silibinin has only been achieved on primary rat hepatocytes, isolated rat Kupffer cells, calves, and mice [37,38,40,41,42].

The in vitro co-culture system may offer suitable alternative to in vivo animal testing and it represents an indispensable tool to approximate the complex conditions in studies aimed at mycotoxin action mechanism in an organism [43]. There have been a few co-culture models used in vitro for studying the absorption of natural bioactive compounds and drug toxicity in hepatocytes [35,43,44,45]. However, this co-culture system was not used to test the efficacy of silibinin in preventing the effects of mycotoxins. In this context, we developed a simple in vitro co-culture model to investigate mycotoxin cytotoxicity on different cell lines. Then, this model was applied to evaluate potential protective effects of silibinin on mycotoxin toxicity. 

The aim of this study was the complex evaluation of toxicity caused by mycotoxins and the possibility of using silibinin to prevent their cytotoxic effect. The toxicity of individual mycotoxins was predicted by in silico analysis and, after that, the data were verified in vitro. To show an additive and synergistic effects of mycotoxin mixtures, the binary mixture was formed by the addition of the second mycotoxin. Silibinin, the predominant compound of the *Silybum marianum*-based dietary supplement, was assessed for its potential protective capacity to prevent toxic effects of mycotoxins in tested cells.

## 2. Results and Discussion

As reported recently, milk thistle-based dietary supplements are usually a significant source of mycotoxins, especially those produced by *Fusarium* and *Alternaria* fungi [46,47]. For some of these mycotoxins, namely DON, HT2, T2, and ZEA, the human health risk has been assessed by European Food Safety Authority (EFSA) [48,49,50,51,52,53] and appropriate maximum limits exist for specific food commodities (1881/2006 EC). For other mycotoxins, such as DAS, ENNs, BEA, MPA, and *Alternaria* toxins, EFSA has not set the tolerable daily intake (TDI) values yet, especially because the relevant toxicity data are still missing, thus the risk assessment process has not been finished. Even though the scientific evidence for heightened toxicity from mycotoxins mixtures is mounting, the risk assessment process, thus the EU legislation, is based predominantly on assessments carried out on individual substances. In this paper, we report on toxicity of specific mycotoxins mixture typical for milk thistle-based preparations, and point to effects resulting from the co-occurrence of these toxins together with silymarin as the most abundant health positive component in this type of foods.

### 2.1. In Silico Prediction of Physicochemical, Pharmacological and Toxicological Properties

The basic physicochemical, pharmacological, and toxicological properties of the mycotoxins previously found in milk thistle-based dietary supplement were evaluated, as summarized in Table 2. Most of the found mycotoxins are soluble in octanol rather than in water. The only hydrophilic mycotoxin is DAS. On the opposite side, BEA is highly lipophilic with the logP value higher than five, which is the limit for bioavailability according to Lipinski’s rule of five [54]. Besides BEA, the octanol–water partition coefficient corresponding to less than one per milliliter of water content was observed for AOH, AME, ENNs, MPA, and ZEA. The very low aqueous solubility of these compounds compromises bioavailability manifested by their poor penetration through the blood–brain barrier and sequestration by fatty tissues. Their poor passive diffusion through the barriers was confirmed by logBB and logPS values for AOH, AME, and MPA followed by high ability to plasma protein binding. In addition, DAS showed poor penetration through the barriers. On the opposite side, ENNs could penetrate and accumulate in CNS.

Several mycotoxins (AOH, AME, and ZEA) showed ability to weakly bind to estrogen receptor (Table 2) and thus affect the endocrine system. From these mycotoxins, only ZEA is predicted to bind to the receptor strongly with the probability of 0.71 and high reliability (RI = 0.88). Genotoxicity was excluded for BEA, ENNs, MPA, TEN, and ZEA by the prediction tool. However, genotoxicity should be expected for AOH, AME, DAS, DON, HT-2, and T-2. The predicted lethal dose for mouse less than 1 ppm was observed for DON, HT-2, and T-2 followed by DAS. The highest lethal dose was found for BEA, which corresponds to its high logP value and low bioavailability. However, BEA is the only mycotoxin that is predicted as a good substrate of P-glycoprotein (*p* = 0.9, RI = 0.38), a transmembrane efflux pump comparable to classical P-gp substrates such as vinblastine, daunorubicin, or paclitaxel. This means that, even though BEA is not able to penetrate through barriers by passive absorption, it uses P-gp pump as the transporter-mediated penetration pathway. DAS, DON, ENNs, HT-2, T-2, and TEN are weaker substrates of P-gp than BEA. PepT1 (intestinal peptide transporter 1), ASBT (intestinal bile acid transporter), or other enzymes were not predicted to be actively involved in transport any of the tested compounds through the intestinal membrane. 

In opposite to logP values, the high permeability via passive absorption across the Caco-2 layer was predicted for AOH, AME, DON, HT-2, and ZEA. The lowest permeability was predicted for DAS.

### 2.2. Verification of the In Silico Prediction

#### 2.2.1. Acute Toxicity of Pure Mycotoxins

The cytotoxic effect of T-2, HT-2, DAS, DON, BEA, ZEA, ENN-A, ENN-A1, ENN-B, ENN-B1, AOH, AME, TEN, and MPA on HepG2, Caco-2, RAW 264.7, and HEK 293T cells was evaluated by resazurin assay over 72 h to determine the mycotoxin concentration that halved the cellular viability (IC_50_). The IC_50_ (nM) values are demonstrated in Table 3. No IC_50_ values were obtained for DON, BEA, ZEA, ENN-A, ENN-A1, ENN-B, ENN-B1, AOH, AME, TEN, and MPA because these toxins did not cause any acute cytotoxicity in a concertation range up to 50 nM. This concentration (50 nM) was chosen intentionally because it was equal to 5× exceeding the recommended daily dose under conditions of 100% bioavailability of the tested compounds. Therefore, this concentration covers absolutely the concentration which could be reached in human blood after oral administration of the supplement. These results are in accordance with observations of Fernández-Blanco et al. [55] studying the cytotoxicity of AME (0–100 µM) in Caco-2 cells. The same result for MPA was reported by Nielsen et al. [56] in human small intestinal cells, where no IC_50_ values were achieved up to 156 nM concentration. Previous studies showed a statistically significant decrease in viability of cells treated at concentrations of BEA (3 µM), DON (1 µM), ZEA (25 µM), ENN-A (1 µM), A1 (1 µM), B (2 µM), B1 (2 µM), AOH (50 µM), and AME (25 µM) [14,19,57,58]. These results are in agreement with those presented in our study, which showed that mycotoxins including DON, BEA, ZEA, ENN-A, ENN-A1, ENN-B, ENN-B1, AOH, AME, TEN, and MPA did not decrease the viability at tested concentrations (up to 50 nM). The highest tested concentration is at least 5× higher than the possible concentration which can be reached by the chosen milk thistle-based dietary supplement (see Section 4.3). It means that even, if the recommended daily dose were exceeded, there would be no risk of acute toxicity of present mycotoxins.

Our results demonstrate that RAW 264.7 cells are extremely sensitive to T-2, HT-2, and DAS, and that T-2 was the most cytotoxic against all tested cell lines, consistent with Behm et al. [59]. These authors assessed the cytotoxicity of 14 mycotoxins in Chinese hamster lung fibroblast (V79 cells) and characterized T-2 as the most potent cytotoxic agent, followed by HT-2 and the other toxins tested. Similarly, previous studies also reported the IC_50_ values for T-2 in the range of 3–500 nM in various mammalian cells [21,56,59,60]. The chemical analysis showed the following concentrations of acutely toxic mycotoxins expected to be in human blood after exposure to the recommended daily dose of milk thistle-based dietary supplement: 6.2 nM of T-2, 5.0 nM of HT-2, and 0.04 nM of DAS. It could be concluded that the doses of T-2 and HT-2 are in the range of IC_50_; however, based on in silico prediction, their penetration through the cell membrane is really low. Therefore, it could be predicted that, after oral administration, neither T-2 nor HT-2 can reach the blood concentration causing the acute toxicity (see also Section 2.4).

The data of in silico and in vitro testing of acute toxicity are in good correlation. In both cases, T-2, HT-2, and DAS were found to be the most toxic mycotoxins. Based on in silico prediction, the doses of all other tested mycotoxins have to be 4-5× higher than those mentioned above, which is over the highest tested concentration as well as the concentration which can be reached in blood by milk thistle-based dietary supplement consumption. The only exception is DON, which was predicted to be as toxic as T-2 and HT-2 mycotoxins, but this was disproved by our in vitro measurements. The prediction was based on structural similarities of DON and T-2, HT-2, and DAS, belonging to the same group of trichothecenes characterized by the tetracyclic 12,13-epoxy trichothecene skeleton [61]. However, in contrast to the others, DON belongs to the Type B trichothecenes, which lack a carbonyl group at C-8 and hydroxyl group at C-7 [62]. Therefore, DON may be less toxic than other trichothecenes such as T-2 toxin. Sobrova et al. [63] published, in agreement with our results, that LD_50_ for mice is several times higher (ranges from 46 to 78 mg/kg after oral administration) than the predicted value.

#### 2.2.2. Genotoxicity of Single Mycotoxins

**Figure 1:**
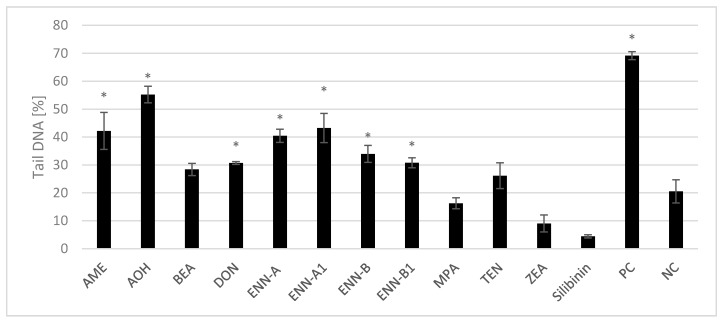
Percentage of DNA in comet tails measured by Comet assay in HEK 293T cells after the treatment with mycotoxins (25 µM) or silibinin (25 µM). Values are expressed as the mean ± SEM (n = 4). *p ≤ 0.05 indicates significant differences when compared to negative control (NC). AME, alternariol-9-methyl ether; AOH, alternariol; BEA, beauvericin; DON, deoxynivalenol; ENN A, enniatin A; ENN A1, enniatin A1; ENN B, enniatin B; ENN B1, enniatin B1; MPA, mycophenolic acid; TEN, tentoxin; ZEA, zearalenone.

Comet assay, widely accepted to evaluate the genotoxic potential of many mycotoxins [64], was used also in our study, where DNA strand breaks in HEK293 cells exposed to mycotoxins (25 µM) were evaluated after 24 h treatment. The results demonstrate that incubation with AME, AOH, DON, and ENNs significantly increased the percentage of DNA in comet tail with respect to the negative control. However, no significant DNA damage was observed in the cells treated with BEA, MPA, TEN, and ZEA (Figure 1).

Genotoxic properties of AOH and AME (25 µM) in HEK293T cells were confirmed by Comet assay where comet tails were about 55% and 42%, respectively. Previous work showed that AOH and AME significantly increased the rate of DNA breaks in human colon adenocarcinoma HT29 cells at concentrations ≥ 1 µM [65,66]. Fehr et al. [64] reported that AOH and AME potently bind to the minor groove of the DNA and act as topoisomerase inhibitors, which might also contribute to the DNA-damaging properties. Moreover, Tiessen et al. [66] indicated that oxidative stress does not play a predominant role in the induction of DNA damage by AOH and AME in HT29 cells. 

The DON genotoxicity reported by Bonny et al. [67] in HepG2 and Caco-2 cells was consistent with our results. The DNA in comet tail increased significantly according to DON concentration ranging from 0.01 to 0.5 µM and the DNA breaks could be explained by DON genotoxicity partly related to the production of free radicals and ROS [67]. In contrast, in the study of Takakura et al. [68], DON (25 µM) failed to induce genotoxicity in human lymphoblast, thymidine kinase heterozygote TK6, and human hepatic HepaRG cells. This study showed that DON induced cytotoxicity without inducing primary DNA damage [68]. The differences may be due to variations among the used cell lines and lower sensitivity to oxidative stress of these cells compared to the HEK 293T cells [69].

Similar DNA damage was observed after 24 h exposure of PK15 and Caco-2 to BEA (0.5 and 12 µM, respectively) [70,71]. Nevertheless, no significant DNA damage was observed in the cells treated with BEA in our study. A similar result was obtained for BEA in the research of Dornetshuber et al. [72]. BEA significantly increased by 85% of DNA in tail with respect to the control at 1 µM but not at 5 µM after 24 h exposure. The reason is that, after 5 µM BEA exposure, antioxidant defense system activities were stimulated and therefore highly contributed to eliminate cell damage. Moreover, BEA inhibited the proliferation of damaged cells arresting them in G0/G1 phase and thus increased the apoptosis [73].

Regarding genotoxicity of ENNs, Prosperini et al. [74] reported significant DNA damage observed for ENN-A (1.5 µM), ENN-A1 (3 µM), and ENN-B1 (3 µM), whereas 3 µM ENN-B did not show genotoxic effect. This may be due to the lipophilicity of the ENNs with the most hydrophobic ENN-A and the least ENN-B (ENN-A > ENN-A1 > ENN-B1 > ENN-B). Moreover, increased ROS generation and lipid peroxidation was observed for all ENNs [74]. 

ZEA-induced oxidative effect on Chang liver cells was evaluated by Kang et al. [75], who found that growing concentrations of ZEA (50–200 μM) increased the DNA damage (from 7.43 ± 0.35% to 19.01 ± 0.42%) [75]. Other authors revealed that ZEA and its metabolites induced oxidative stress by increasing the level of ROS, which can cause damage to DNA [76]. Gao et al. [77] found that ZEA (2.5–20 µM, for 2 h) damaged DNA in HEK293 cells in a concentration-dependent manner. However, the results suggest lysosomes disruption rather than oxidative stress plays a key role in DNA strand breaks induced by ZEA. Therefore, the authors predicted the lysosomes as a primary target of ZEA [77]. On the contrary, we found that HEK 293T cells were resistant to the treatment with 25 µM of ZEA for 24 h. These results suggest activation of DNA repair mechanisms in the cells upon prolonged incubation [78].

#### 2.2.3. P-gp Substrate Probability of Single Mycotoxins

**Figure 2:**
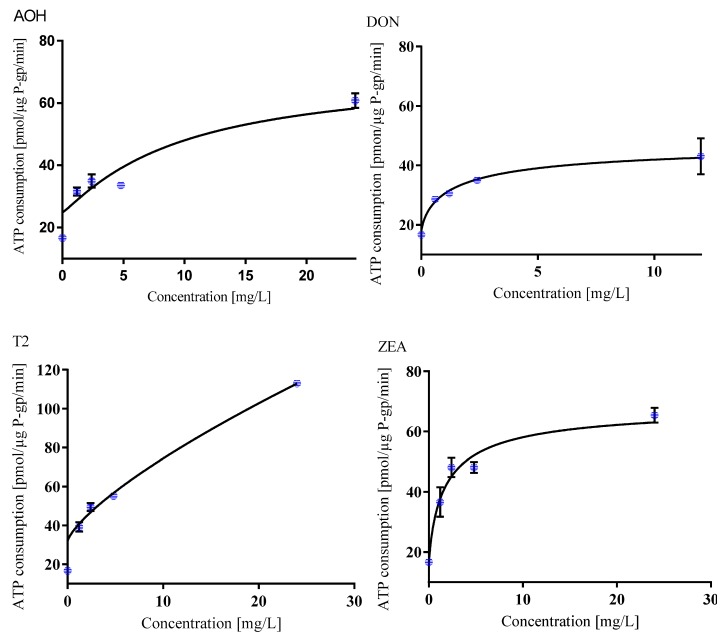
Mycotoxins as substrates of P-gp: AOH; DON; T2; and ZEA. Values are expressed as the mean ± SEM (n = 3). AOH, alternariol; DON, deoxynivalenol; T-2, T-2 toxin; ZEA, zearalenone.

P-glycoprotein (P-gp), which is also known as multidrug resistance protein 1 (MDR1) or ATP-binding cassette sub-family member B 1 (ABCB1), is an ATP-dependent efflux pump transporting a wide range of hydrophobic compounds including drugs and other xenobiotics [79]. It limits the drug entry into the body, promotes drugs elimination into bile and urine, and decreases drug penetration into sensitive tissues [80]. For the in vitro evaluation of whether mycotoxins serve as P-gp substrates, an isolated fraction of the P-gp enriched membranes was used. As P-gp activation is coupled with ATP consumption, we measured the in vitro ATP consumption reflecting the transport activity [81]. From the whole spectrum of mycotoxins, only AOH, DON, T-2, and ZEA activated P-gp in dose-dependent manner. T-2 toxin caused the most significant increase in ATP consumption, suggesting its P-gp-mediated transport (Figure 2).

Li et al. [82] and Videman et al. [83] proposed that P-gp is the foremost transporter of DON in Caco-2, HepG2, and MDCK cells. Different models indicate that P-gp is directly involved in the efflux of ZEA [84,85,86]. It was reported that T-2 is not a substrate of P-gp [87]. However, in our study, ATP consumption indicated the highest P-gp substrate probability of T-2 followed by ZEA, AOH, and DON. Even though T-2 and AOH are transported via isolated P-gp in our in vitro model, in the cells, the situation is more complex and P-gp efflux activity may be inhibited by various mechanisms or the mycotoxins could be metabolized or substituted.

### 2.3. Influence of the Mycotoxins Properties in Mixtures

In the above sections, we summarize the toxicity of single mycotoxins, but, in natural resources, they predominantly occur in mixtures. Mixtures of mycotoxins can be present in materials of natural origin (e.g., food, feed, and dietary supplements) because: (i) the material can be contaminated by several molds; (ii) some molds can produce several different mycotoxins; and (iii) the material is prepared from several contaminated plants or plant species [88]. Based on our analysis, the milk-thistle dietary supplement may be contaminated by up to 14 different mycotoxins originating from several molds. As the limits of detection were very low, we addressed the question whether the detected levels of mycotoxins are toxic to human cells.

#### 2.3.1. Determination of Acute Toxicity of Milk Thistle-Based Dietary Supplement

**Figure 3:**
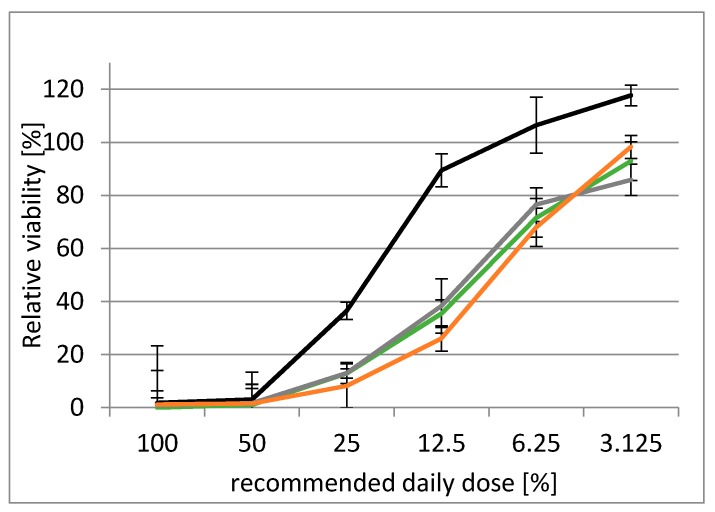
HEK 293T cytotoxicity of milk thistle-based dietary supplement and the model mixtures of toxins. The black line is a mixture mimicking the mycotoxins occurrence in the supplement, the green line is a mixture mimicking the silymarin composition, the grey line is a mixture mimicking the mycotoxins plus silymarin composition, and the orange line is a milk thistle-based dietary supplement.

Several mycotoxins model mixtures mimicking the milk thistle-based dietary supplement were prepared to determine their cytotoxic effects. These mixtures were mimicking: (i) the concentration and composition of mycotoxins in particular supplement; (ii) the concentration and composition of silymarin; and (iii) the concentration and composition of mycotoxins and silymarin in the supplement. The cytotoxicity of these model mixtures was compared with the cytotoxicity of milk thistle-based dietary supplement using human embryonal kidney cells (HEK 293T). As can be seen in Figure 3, all tested mixtures completely inhibited the cell viability at concentrations corresponding to 50–70% of recommended daily dose (100% recommended daily dose was equal to 8 nM of ALT, 4.5 nM of AME, 9.4 nM of DON, 6.2 nM of T-2, 5.0 nM of HT-2, 0.04 nM of DAS, 1.1 nM of ZEA, 3.7 nM of TEN, 3.5 nM of BEA, 0.7 nM of ENN-A, 1.7 nM of of ENN-A1, 2.7 nM ENN-B, 2.6 nM of ENN-B1, and 29 µM of silibinin). No toxic effect, manifested by zero effect on the cell viability, was observed at concentrations lower than 3% of recommended daily dose.

The toxicity of silibinin, the main component of silymarin complex, has been previously published many times for such high doses as we tested (100% recommended daily dose was equal to 29 µM concentration of silibinin) [89]. The concentration of silymarin composition in recommended daily dose exceeds by one order of magnitude that of the mycotoxins occurring at considerably lower concentrations, which did not affect the acute cytotoxicity (Table 4). Even though fourteen different mycotoxins were present in the mixture, single mycotoxins can vary in their modes of action. Therefore, their combination may dramatically affect the cellular processes by an additive or synergistic effect.

#### 2.3.2. Acute Toxicity of Mycotoxins in Binary Mixtures

To investigate the type of interactions between selected mycotoxins in binary combinations, T-2, HT-2, and DAS were applied in IC_50_ doses in a mixture with another mycotoxin in a 1:1 ratio. At these concentration, T-2, HT-2, and DAS caused 50% cell mortality, while the others did not significantly reduce the cell viability.

The binary combinations lowered cell viability compared to single compounds. The T-2 and DAS mixture (DAS+T-2) as well as their combination with HT-2 (HT-2+T-2 and HT-2+DAS) and some others (ENN A1+DAS and MPA+DAS) significantly reduced cell viability by about 31%, 33%, 24%, 16%, and 15%, respectively. In contrast, cell viability was not significantly reduced by other combinations of toxins (mixtures containing DON, ZEA, ENN-A, ENN-B, ENN-B1, TEN, BEA, AOH, and AME, as well as ENN-A1+T-2, ENN-A1+DAS, MPA+T-2, and MPA+DAS) (data not shown). 

To determine the mode of action, the binary mycotoxin interactions were further assessed by the conceptual model called a “linear interaction effect”. Table 5 indicates additive effect for the binary mixtures of T-2 with ZEA, BEA, AOH, or AME. Similarly, additive effect was demonstrated for the binary mixtures of DAS with TEN, BEA, AOH, or AME and also for the binary mixtures of HT-2 with ENN-A1, ENN-B1, TEN, MPA, BEA, AOH, or AME. Summarizing the results, each binary combination of T-2, DAS, and HT-2 with TEN, BEA, AOH, or AME had an additive effect. The binary mixture of BEA and T-2 has been previously demonstrated as antagonistic in Vero cells [23] while synergistic in CHO-K1 cells [14].

We found synergistic effects in the binary mixtures of T-2 with ENN-A, ENN-B1, MPA, HT-2, or DAS. A synergistic effect of DAS was also observed when mixed together with either HT-2, DON, ZEA, ENNs, or MPA and for HT-2 combined with DON, ZEA, ENN-A, or ENN-B in a binary mixture. Summarizing the results, ZEA, ENNs, and MPA caused either a synergistic or at least an additive effect except two combinations (T2 with ENN-A1 or ENN-B) where an antagonistic effect was observed.

The observed additive or synergistic effect for combinations with ENNs, MPA, BEA, AOH, or AME could be explained (based on Table 1) as follows: BEA and ENNs increase the cell permeability and thus make the cells more accessible for the other mycotoxins acting as ionophores. Similarly, MPA, AOH, and AME decrease the cell proliferation, which in general decreases cell viability.

A synergistic effect might be caused by the fact that mycotoxins influence different stages of the same toxicity pathway by increased absorption or decreased metabolic degradation of one mycotoxin at the presence of another one [90]. The synergistic effect of T-2 in combination with DAS demonstrated in this study is in agreement with the results of Thuvander et al. [91].

In contrast, an antagonistic effect was demonstrated in the combination of T-2 with DON, ENN-A1, or ENN-B. Several previous studies are consistent with our results describing the interaction between DON and T-2 in Vero cells [14], in CHO-K1 cells [14], and in human lymphocytes [91]. A similar result was obtained by Fernadez-Blanco et al. [14,19] who found that the combination of DON and ENN-B resulted in the antagonistic interaction. The authors explained this interaction by the competition between the mycotoxins for the same target/receptor site [14,19]. Lin et al. [92] suggested that the effect of DON and T-2 is additive/synergistic at middle and high concentrations but antagonistic at low concentrations in rat chondrocytes and C-28/I2 cells. Additionally, synergic effect was also detected when the individual levels were at nearly the same ratio and antagonistic effect when the concentration of DON was much higher (100-1000×) than T-2 [92].

In general, additive, antagonistic, and synergistic effects depend not only on the compounds in the mixture, but also on their mutual concentrations and exposure time [92,93].

#### 2.3.3. Suppression of Mycotoxins´ Acute Toxicity by Silibinin

The IC_50_ of T-2, HT-2, and DAS halving the HEK293T cells viability after 24 h of incubation were determined as 7, 30, and 15 nM, respectively. At these concentrations, silibinin at the concentration range from 0.9 to 109 µM was added to evaluate its preventive effect in extended range of recommended daily dose of the supplement. As previously published, the co-treatment with both silibinin and mycotoxins did not improve the cell growth, but pretreatment of the cells by silibinin for 2 h before mycotoxins addition protected the cells against mycotoxins-mediated apoptosis [41,42]. Thus, we chose this approach to evaluate the protective effect of silibinin against mycotoxins-induced cytotoxicity.

The presence of silibinin alleviated the HEK 293T viability decrease caused by single mycotoxins (T-2, HT-2, and DAS) in a concentration-dependent manner. The pretreatment with silibinin at lower concentrations (below 54.5 µM) had almost no effect on the cell viability. The highest tested concentration of silibinin (109 µM) showed an additive toxic effect resulting in about 30% decrease of the cell viability when compared to viability of cells cultured with a single mycotoxin. Regarding the effect of the sole silibinin treatment on the cell viability, no toxicity was shown after 24 h of exposure, except for the highest concentration point (109 µM), which significantly reduced the cell viability by 45%. It has been already reported that silibinin had no significant cytotoxic effect on human fibroblasts at the 50 µM concentration for 24 h [94].

Due to the different effect of silibinin pretreatment on the HEK 293T cells cultivated with toxic doses of T-2, HT-2, and DAS, the type of interaction between silibinin and mycotoxins was evaluated by the “linear interaction effect” model (see Table 6). The strongest antagonism was found with the silibinin pretreatments at concentration of 13.6 µM for T-2 and 6.8 µM for HT-2 and DAS exposure,. The cytoprotective effect of silibinin, may be ascribed to its antioxidant and free-radical scavenger role [41]. Based on the results of Al-Anati et al. [40], low silibinin dose (0.2 µM) reduced OTA-induced TNF-α level to 70% while the higher doses (1–26 µM) completely blocked OTA-induced TNF-α within 24 h. Furthermore, the protective effect of 130 µM silibinin against OTA cytotoxicity in hepatocyte cells was reported [41,42]. The authors assumed that silibinin acts on the cell membranes to prevent the entry of toxic substances, stimulates protein synthesis, and accelerates regeneration processes. According to Fan et al. [95], silibinin activated p53 in a dose-dependent manner and thus induced ROS generation in HeLa cells. Therefore, there is evidence of silibinin’s pro-oxidative action [36]. However, silibinin could not induce ROS generation in A431 cells without normally functioning p53 [95]. In this cell line, silibinin did not trigger ROS generation but scavenged ROS.

Similarly, a silibinin-induced cell death in human breast cancer cell lines MCF7 and MDA-MB-231 was dependent on ROS generation [96]. Similar to silibinin, trichothecenes also generate free radicals resulting in lipid peroxidation with changes in the membrane integrity, cellular redox signaling, and overall redox status [97]. In this study, high doses of silibinin (55-109 µM) caused cytotoxic effect when HEK293T cells were exposed to T-2, HT-2, and DAS. In this case, it could be hypothesized that the additivity and slight antagonism are the sum of individual effects of silibinin and mycotoxins. Consequently, antioxidant and pro-oxidant effects of silibinin are largely related to its concentration in a given biological system. Duan et al. [98] suggested that silibinin can possess both survival and death effects depending on the dose and time of exposure.

#### 2.3.4. Protective Effects of Silibinin Against Mycotoxin Genotoxicity

**Figure 4:**
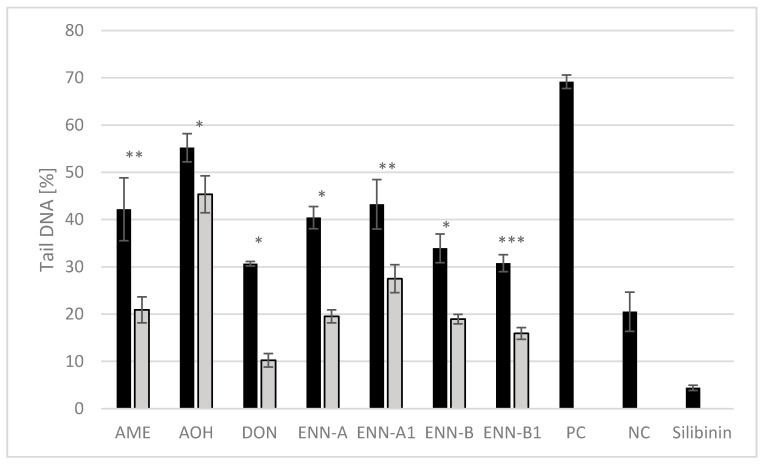
Inhibitory effect of silibinin on DNA damage induced by mycotoxins in HEK 293T cells. Values are expressed as the mean ± SEM (n = 4). Stars indicate significant differences between silibinin-treated (grey columns) and silibinin-untreated (black columns) comets caused by mycotoxins: *p ≤ 0.05, **p ≤ 0.005, ***p ≤ 0.0005. AME, alternariol-9-methyl ether; AOH, alternariol; DON, deoxynivalenol; ENN A, enniatin A; ENN A1, enniatin A1; ENN B, enniatin B; ENN B1, enniatin B1.

The trend of DNA damage induced by AME, AOH, DON, or ENNs in HEK 293T cells was significantly decreased by the addition of 25 µM silibinin (Figure 4). Silibinin at the concentration of 25 µM did not induce genotoxic effects in HEK 293T cells and silibinin treatment attenuated the mycotoxin-induced DNA damage indicating its anticlastogenic potential. Abdel-Wahhab et al. [99] reported that treatment with silymarin nanoparticles protected the liver against hepatic oxidative stress, genotoxicity, and cytotoxicity of DON in rats. In the study of Togay et al. [100], DNA damage in rats with streptozotocin-induced diabetes symptoms were decreased after silibinin treatment. Similarly, Fernandes Veloso Borges et al. [101] showed that silibinin (5.2–15.5 mM) reduced the amount of DNA in the comet tail compared to positive control. Silibinin is known to exhibit strong antioxidant activities and its protective effects against ROS have been demonstrated in different cell lines [41,42]. Protective effects of silibinin on DNA can be explained by scavenging of ROS [102,103]. DON- and ENNs-induced genotoxicity may be associated with the oxidative stress [67,74], while oxidative stress does not play a predominant role in the induction of DNA damage by AOH and AME [66]. This could explain why the treatment with silibinin caused formation of smaller DNA tails induced by DON and ENNs compared to AOH and AME.

### 2.4. Bioavailability of Mycotoxins and Silibinin

Upon ingestion, mycotoxins can be degraded or modified by biotransformation in the intestinal mucosal wall and only a fraction of the initial content can be absorbed from the gut via intestinal cells [44,104,105]. In this sense, bioavailability is a term used to describe the portion of ingested contaminant that reaches the bloodstream [106]. In the present study, we evaluated the transport of mycotoxins and silibinin by using a two-compartment transwell system representing a co-culture of Caco-2 and RAW 264.7 cells. 

Based on the results in Table 7, the bioavailability of silibinin and mycotoxins were not significantly different, especially in the systems treated with AME, DAS, and ZEA. For most of the mycotoxins and silibinin, low aqueous solubility was predicted, which could limit their bioavailability. Previous publications showed that DON, T-2, and HT-2 were transported efficiently through the epithelial cell layers with up to 38% after 24 h, 24% after 6 h, and 32% after 24 h, respectively [107]. ZEA was efficiently absorbed and α-ZEA and β-ZEA are the two major metabolites produced by Caco-2 cells (41% and 32% of total metabolites, respectively; after 3 h exposure to 10 µM ZEA) [108]. BEA bioavailability was 50% and 54% after 4 h exposure to 3 and 4.5 µM of this mycotoxin, respectively [71].

In our study, the bioavailability of ENNs was similar. However, ENN-A and A1 were detected in higher amount in cells while ENN-B and B1 had higher concentration in basolateral medium. A similar result was also obtained in study by Meca et al. [109]; ENN-B and B1 were more bioavailable from the lumen to blood in the Caco-2 cells compared to ENN-A and A1. Because the total amounts of mycotoxins AME, AOH, DAS, ENN-B, and TEN in the system were significantly lower than their original amounts added into the apical medium, we can presume their metabolization. This fact was also confirmed by our in silico prediction as well as by non-target U-HPLC-MS analysis. By this analysis, we confirmed the presence of AME metabolites (3-O-glucuronide, 7-O-glucuronide, and 3-O-sulfate), AOH metabolites (3-O-glucuronide and 9-O-glucuronide), DAS metabolites (15-monoacetoxyscirpenol, 4-monoacetoxyscirpenol, 7-hydroxy, 8β-hydroxy, and deepoxy-15- monoacetoxyscirpenol), ENN-B metabolite (metabolite M6), and TEN metabolites (metabolite M1, metabolite M2, and metabolite M3). These metabolites were previously described in several studies [110,111,112,113]. 

Based on the total amount of mycotoxins found in our transepithelial system, BEA, ENN-A, A1, B1, T-2, and silibinin should be metabolized as well. T-2 metabolites were detected in our system, namely 3´-hydroxy, 3-hydroxy-15-deacetyl as well as its main metabolite, and HT-2 derivatives (3´-hydroxy-HT-2, 4′-hydroxy-HT-2, deepoxy-3′,7-dihydroxy-HT-2 [111]). The main silibinin metabolite found was a sulfate derivative. In human cells, T-2 could mainly transform to HT-2 and nesolaniol or other products such as 3′-hydroxy-T-2, 4-deacetylnesolaniol, T-2 glucuronide, and HT-2-glucuronide [97]. 

HT-2 and DON were dominantly detected, mostly in apical medium. This might be because DON was not metabolized by intestinal cells and HT-2 was the main metabolite of T-2, which was previously observed [107]. Besides, BEA and enniatins were found in both RAW 264.7 and Caco-2 cell fractions. It seems that these mycotoxins interact with the membrane according to their in silico predicted lipophilicity.

In agreement with in silico prediction, MPA was unavailable to the cells thanks to its low solubility.

## 3. Conclusions

In the present study, ACD/Percepta was used to predict the properties of mycotoxins and silibinin. In comparison to in silico prediction, in vitro cytotoxicity studies confirmed that T-2, HT-2, and DAS exhibited the highest cytotoxicity of the fourteen mycotoxins tested. The binary combination results suggest that the co-occurrence of these mycotoxins may increase their cytotoxic effects compared to a single mycotoxin. The findings on protective effects of silibinin against both the acute cytotoxicity of mycotoxins (T-2, HT-2, and DAS) and genotoxicity of AME, AOH, DON, and ENNs on HEK 293T in a dose-dependent manner should be taken into account. Finally, the bioavailability of mycotoxins and silibinin does not differ too much and most of the them are metabolized during the transport through epithelial cell layer.

## 4. Materials and Methods 

### 4.1. Reagents and Instrumentations

The following chemical reagents and cell culture components were purchased from Sigma-Aldrich (USA): Dulbecco’s Modified Eagle’s Medium (DMEM), Minimum Essential Medium (MEM), trypsin/EDTA solutions, antibiotic mixture (penicillin and streptomycin), phosphate buffer saline (PBS), resazurin sodium salt, silymarin, silibinin and mycotoxins. Stock solutions of T-2, HT-2, DAS, DON, BEA, ZEA, ENN-A, ENN-A1, ENN-B, ANN-B1, AOH, AME, TEN, and MPA were prepared in methanol and maintained at -20 °C in dark. The final concentrations of methanol in the solutions of mycotoxins in culture medium were ≤ 1% (v/v).

### 4.2. Cell lines and Cell Cultures

Human colon adenocarcinoma (Caco-2), mouse macrophage (RAW 264.7), human hepatoblastoma (HepG2), and embryonic kidney (HEK 293T) cell lines were obtained from ATCC (USA). Stock cultures of RAW 264.7 and HEK 293T cells were maintained in DMEM, while Caco-2 and HepG2 cells were cultured in EMEM. Both media were supplemented with fetal bovine serum (FBS) (10%) and 1% of antibiotic mixture (penicillin, 100 IU/mL and streptomycin, 100 g/mL) incubated at 37 °C in the atmosphere of 5% CO2. For cell counting and subculture, the cells were dispersed with a solution of 0.05% trypsin and 0.02% EDTA. The medium was changed every third day, and the cells were passaged at approximately 80% confluence. At passages 9–29, the cells were seeded in 96-well plates for the cytotoxicity assays and, passages 30–50 of the Caco-2 cells were used for the co-culture system.

### 4.3. Milk Thistle-based Dietary Supplement 

The milk thistle-based dietary supplement (Ostropestřec plus, Farmax^®^, Ruakura, New Zealand) was purchased on the Czech market. Its characterization, as provided by manufacturers, was as follows: milk thistle extract (*Silybum marianum*, seed) 250 mg in one capsule—standardized to contain 80% silymarin. The internal content of twenty capsules was weighed separately and then mixed together to obtain the homogenized representative sample. 

The quantitative analysis, which was published previously [46], showed total content of mycotoxins equal to 4 ng in one capsule. This content was recalculated according to recommended daily dose and the volume of blood in the human body (5 L) as follows: 8 nM AOH, 4.5 nM AME, 9.4 nM DON, 6.2 nM T-2, 5.0 nM HT-2, 0.04 nM DAS, 1.1 nM ZEA, 3.7 nM TEN, 3.5 nM BEA, 0.7 nM ENN-A, 1.7 nM ENN-A1, 2.7 nM ENN-B, and 2.6 nM ENN-B1. The concentrations of silibinin, the most abundant component of silymarin complex, was 29 µM in the supplement.

### 4.4. In Silico Toxicity Analysis

ACD/Percepta (ACD/Percepta Platform, version 2016, build 2911, Advanced Chemistry Development, Inc., Toronto, ON, Canada, www.acdlabs.com, 2016) was used to predict the most common physicochemical, pharmacokinetic and toxicology properties. For ACD/Percepta data, a reliability index (RI) higher than 0.75 was considered as highly reliable (marked as a), while RI < 0.5 was considered as borderline reliable (marked as *).

### 4.5. Cytotoxicity Assay

The cells were counted by Cellometer Auto T4 (Nexcelom Bioscience, Lawrence, MA) and the cell suspension containing cell density 10^5^ cells/mL was split into the 96-well plate. The plates were then incubated for 24 h at 37 °C in humidified atmosphere of 5% CO_2_. Then, the tested compounds were added. To assess the effect of silibinin, cells were pre-treated with silibinin at the given concentrations 2 h prior to mycotoxin exposure. After 72 h incubation, the cell viability was tested by standard resazurin assay [114]. Briefly, the cells were washed three times with 100 µL of PBS and incubated with 100 μL of resazurin solution (0.025 mg/mL) for 3 h. Finally, the fluorescence was measured by a SpectraMax i3x microplate reader (Molecular Devices, UK) at a wavelength of 560 nm excitation/590 nm emission.

### 4.6. P-gp Substrate Determination

The in vitro P-gp activation was tested using the Pgp-Glo Assay System according to the standard procedure [79]. Briefly, the reaction mixture contained Pgp-Glo Assay buffer, P-gp containing membranes, and MgATP in a total volume of 50 µL. As the controls, Na_3_VO_4_ (P-gp inhibitor) and verapamil (P-gp substrate) were used. Mycotoxins were added and the reaction mixture was incubated for 1 h in 37 °C. The reaction was stopped by the addition of the detection reagent (50 µL). After 20 min of incubation, the luminescence was recorded.

The luminescence (ΔRLU samples) was calculated as the difference between the relative luminescence of Na_3_VO_4_ and that of the samples. For the P-gp substrates, the specific activity of P-gp was determined using the standard ATP curve and calculating the amount of nanomoles of ATP consumed per µg of P-gp per minute. The standard ATP curve was determined by linear regression and the concentrations of ATP consumed in the samples were recalculated by the subsequent standard interpolation of RLU ATP.

### 4.7. Caco-2/RAW 264.7 Co-Culture System

Caco-2 cells were re-seeded in polycarbonate membrane inserts (0.4 μm pore diameter, 12 mm insert 12-well plate; Corning, USA) at 2 × 10^5^ cells/cm^2^ [43]. The culture medium was changed three times a week. After 21 days post-seeding, the monolayers of differentiated Caco-2 cells were used for co-culture experiments. Only the monolayers expressing a transepithelial flux of phenol red to the basolateral compartment of approximately 10^−7^ cm/s [115] were used in subsequent experiments. For the co-culture system, RAW 264.7 cells were seeded in 12-wells plates at a density of 8.5 × 10^5^ cells/well (at Day 20 of the Caco-2 cells differentiated monolayers) [116]. Twenty-four hours after seeding of RAW 264.7 cells, the co-cultures were performed (Day 21). Non-cytotoxic doses of mycotoxins were selected for 4 h treatment in transwell plates.

### 4.8. U-HPLC-MS Determination of Mycotoxins and Silibinin

All culture medium from apical, basolateral compartments, Caco-2 and RAW 264.7 cells were collected after treatments. Mycotoxins and silibinin were extracted by ethanol and the extracts were analyzed by U-HPLC-MS according to previously described instrumental method [46]. The concentration of mycotoxins and silibinin were determined by external calibration batch of analytical standards dissolved in ethanol to concentration range of 0.1–200 ng/mL for mycotoxins and 50–2500 ng/mL of silibinin. The repeatability of the method, expressed as relative standard deviation (RSD), was evaluated by repeated analysis (n = 7) of control samples (both culture media and cell types) fortified before extraction by mycotoxins standard mixture and silibinin to final concentration in extract of 50 and 500 ng/mL, respectively. The RSD were in the range of 1.5–7.3% for all of the analyte/matrix combinations. The limits of quantitation (LOQ), evaluated for each of the analytes as the lowest level of calibration batch laying within linear concentration range, were 0.1–0.5 ng/mL for particular mycotoxins and 50 ng/mL for silibinin.

### 4.9. Combination Effect of Mycotoxins in Binary Mixtures

The “linear interaction effect”, also called “response additivity”, was used to evaluate the mycotoxins combined effects [43]. A combination index (CI) was calculated for each combination. This index is recognized as a standard measure of combination effect and CI < 0.9, 0.9 ≤ CI ≤ 1.1, and CI > 1.1 indicate synergism, additivity, and antagonism, respectively. The CI of “linear interaction effect” model can be calculated as:

(1) $$CI=\frac{observed\;effect\;\left(mycotoxin\;1\right)+observed\;effect\;\left(mycotoxin\;2\right)}{observed\;effect\;\left(mycotoxin\;1+mycotoxin\;2\right)}$$

### 4.10. Genotoxicity Assay

Genotoxicity was studied by standardized method known as a single cell gel electrophoresis (Comet assay) [117]. Briefly, HEK 293T cells were seeded in 12-well plates (10^6^ cells/mL) and treated with mycotoxins (25 µM) or combination of mycotoxin (25 µM) and silibinin (25 µM) for 24 h. 

DMSO was added to control cells in the concentration of 0.6%, which is identical to the concentration of the solvent in the tested samples. Cells for the positive control were treated with 100 μM H_2_O_2_ in PBS for 10 min in 37 °C.

The cells were detached using trypsin (EDTA) and frozen in freezing medium (10% DMSO, 40% serum, 50% DMEM) at -160 °C. For the Comet assay, the cell suspension was centrifuged at 1000 × *g* for 1 min. The pellet was resuspended in 0.5 mL of PBS. A 50-μL aliquot of the suspension was mixed with 150 μL of LMP agarose (0.01 g/mL). Then, 80 μL of resulting agarose–cell suspension were spread onto the slide pre-coated with 1% regular agarose. The slides were allowed to solidify followed by cell lysis, gel electrophoresis, and staining [118] Slides were scored using an image analysis software (ImageJ 1.51s, National Institutes of Health, WI, USA) connected to a fluorescence microscope (AX70 Provis, Olympus, Japan). All experiments were performed in tetraplicate, and in each parallel images of 100 randomly selected cells were evaluated. Comet parameters considered in this study were the tail length and the proportion of DNA in the comet tail (tail DNA or tail intensity).

### 4.11. Data Processing and Statistical Analysis

All experiments were independently repeated at least three times (biological replicates). In addition, each replicate included at least three replicated treatments (technical replicate). The relative activity was evaluated as a percentage according to the formula:

(2) $$RA\;\left(\%\right)=100\times \frac{\left(\mathrm{slope}\;\mathrm{of}\;\mathrm{sample}-\mathrm{average}\;\mathrm{slope}\;\mathrm{of}\;\mathrm{PC}\right)}{\left(\mathrm{average}\;\mathrm{slope}\;\mathrm{of}\;\mathrm{NC}-\mathrm{average}\;\mathrm{slope}\;\mathrm{of}\;\mathrm{PC}\right)}$$

The results are expressed as the average ± standard error of the mean (SEM). Values of IC_50_ were obtained by using the software GraphPad Prism 8.0 (GraphPad Software Inc., San Diego, CA, USA) and nonlinear regression:

(3) $$Y=\frac{\mathrm{Bottom}+\left(\mathrm{Top}-\mathrm{Bottom}\right)}{1+10^{\left(logIC_{50}-X\right)\times HillSlope}}$$

The significance was tested by one-way ANOVA followed by the t-test for multiple comparisons using Statgraphics software (Statgraphics Technologies, Inc., USA) and the Excel t-test function (two-tailed distribution, heteroscedastic type). *p*-values < 0.05 were considered statistically significant.

## Figures and Tables

**Table 1:** Toxicity of selected mycotoxins.

Mycotoxins	Effects	References
T-2 and HT-2	Inhibition of DNA, RNA, and protein synthesis. Induction of mutations and apoptosis.	[12,13,14,15]
DAS	Inhibition of DNA and protein synthesis. Suppression of macrophage phagocytic function.	[16,17]
DON	Inhibition of DNA, RNA, and protein synthesis. Decrease of the cell proliferation.	[18,19]
ZEA	Activation of the estrogen receptor. Inhibition of DNA and protein synthesis. Triggering of lipid peroxidation and cell death.	[20,21,22]
BEA	Increase of the biological membrane. Loss of ionic homeostasis. Induction of lipid peroxidation.	[14,23,24]
ENNs	Increase of the membrane permeability for cations.	[25]
AOH and AME	Single and double strand DNA breaks. Decrease of the cell proliferation.	[26,27,28]
TEN	ATP hydrolysis and inhibition of ATP synthesis.	[29]
MPA	Inhibition of inosine 5′-monophosphate dehydrogenase. Blocking of the DNA synthesis and proliferation of both T and B lymphocytes.	[30,31]

**Table 2:** In silico toxicity analysis of mycotoxins previously identified in milk thistle-based dietary supplement.

Parameters		*Fusarium* Toxins										*Alternaria* Toxins			*Penicillium* Toxin	Silibinin
		trichothecenes				others										
		DON	HT-2	T-2	DAS	ZEA	ENN-A	ENN-A1	ENN-B	ENN-B1	BEA	AME	AOH	TEN	MPA	
**octanol–water partition coefficient**	logP	1.5	1.2	2	-0.4	4.1	4.7*	4.4*	3.9*	4.1*	5.9*	3.9	3.8	0.5	3.8	2.1
**BBB (blood–brain barrier) permeability**	logPS	−2.2	−2.0	−2.0	−3.8	−1.4	−1.3	−1.4	−1.8	−1.6	−1.4	−1.6	−1.9	−2.5	−2.9	−2.9
	logBB	0.2	0.6	0.5	−0.07	0.6	1.4	1.0	0.5	0.8	0.3	−0.1	−0.4	−0.2	−0.7	−0.9
**human serum affinity**	LogKa (HSA)					4.0*								3.4*	4.7	
**plasma protein binding**	PPB (%)					88.1						97.0*	95.8	65.7	96.0^a^	98.0
**estrogen receptor binding probability**	Log (RBA) > −3	0.0*	0.0*	0.0	0	0.9	0^a^	0^a^	0^a^	0^a^		0.9	1.0	0		
**genotoxicity probability**	CHO/CHL all loci composite	0.8	0.8	0.7	0.8	0.2	0.0	0.0	0.0	0.0	0.0	0.9	0.9	0.1	0.2	0.2
	chromosomal aberration in vitro	0.7	0.6	0.6	0.9	0.6	0.1	0.1	0.1	0.1	0.1	0.6	0.8	0.4	0.6	0.5
	chromosomal aberration *in vivo*	0.6	0.5	0.5	0.8	0.2	0.1	0.1	0.1	0.1	0.1	0.6	0.7	0.4	0.2	0.5
	carcinogenicity in mice	0.4	0.3	0.3	0.4	0.7	0.1	0.1	0.1	0.1	0.1	0.4	0.4	0.2	0.3	0.2
**LD_50_ for mouse**	(mg/kg)	0.8	0.8^a^	0.9	1.2	3.3*	3.0	3.0	3.0	3.0	3.7		3.4*	3.0	3.2	
**P-gp substrate**	substrate probability	0.8	0.9	0.9	0.8*	0.2*	1.0	1.0	0.9	0.9	1.0*		0.1	0.8*	0.4	0.2*
**Caco-2 permeability**	Pe (10 ^−4^ cm/s)	7.2	7.0	7.0	0.2	7.8	6.0	6.0	6.1	6.1	5.8	8.6	8.4	6.0	4.7	5.1
**first pass metabolism**		N	N	N	Y	N	N	N	Y/N	N	N	Y	Y	Y	Y	

ACD/Percepta (ACD/Percepta Platform, version 2016, build 2911, Advanced Chemistry Development, Inc., Toronto, ON, Canada, www.acdlabs.com, 2016) was used to predict the most common physicochemical, pharmacokinetic, and toxicology properties. For ACD/Percepta data, a reliability index (RI) higher than 0.75 was considered as highly reliable (marked as ^a^); RI < 0.5 was considered as borderline reliable (marked as *). AME, alternariol-9-methyl ether; AOH, alternariol; BEA, beauvericin; DAS, diacetoxyscirpenol; DON, deoxynivalenol; ENN A, enniatin A; ENN A1, enniatin A1; ENN B, enniatin B; ENN B1, enniatin B1; HT-2, HT-2 toxin; MPA, mycophenolic acid; T-2, T-2 toxin; TEN, tentoxin; ZEA, zearalenone.

**Table 3:** IC_50_ of T-2, HT-2, and DAS on HepG2, Caco-2, HEK293T, and RAW 264.7 cell lines. Data are expressed as mean values ± SEM of independent experiments (n = 3), each with six technical replicates.

Mycotoxins (nM)	Cell lines			
	RAW 264.7	Caco-2	HepG2	HEK293T
T-2	3.57 ± 0.27	13.37 ± 1.07	11.38 ± 0.37	3.87 ± 0.27
HT-2	5.07 ± 0.46	44.23 ± 2.26	47.44 ± 1.29	21.22 ± 1.6
DAS	3.66 ± 0.37	17.74 ± 0.66	13.4 ± 1.79	6.58 ± 0.36

DAS, diacetoxyscirpenol; HT-2, HT-2 toxin; T-2, T-2 toxin.

**Table 4:** Cytotoxicity of the milk thistle-based dietary supplements and the model mixtures. Cytotoxicity was evaluated by the Duncan´s post hoc analysis expressing the differences between the groups. Similar or sharing letters within one concentration (e.g., a and ab) show that there are no significant differences in cell viability of HEK 293T cells treated with milk thistle-based dietary supplements and the model mixtures. Different letters (e.g., a and b) show the statistical difference (*p* ≤ 0.05) between milk thistle-based dietary supplements and the model mixtures within the tested concentration.

Tested Mixtures	Recommended Daily Dose (%)					
	**100**	**50**	**25**	**12.5**	6.25	3.13
**Silymarin**	a	a	a	a	a	a
**Mycotoxins plus silymarin**	a	a	a	a	a	a
**Milk thistle-based dietary supplement**	a	a	a	a	a	a,b
**Mycotoxins**	a	a	b	b	b	b

**Table 5:** The combination indexes of binary mycotoxin mixtures on HEK293T after 72 h exposure. The indexes report on mechanism of combined exposition. T-2, HT-2, and DAS were applied in IC_50_ doses in a mixture with another mycotoxin in a 1:1 ratio.

Mycotoxins	DAS	HT-2	DON	ZEA	ENN A	ENN A1	ENN B	ENN B1	TEN	MPA	BEA	AOH	AME
Mixture of T-2 (3.87 nM)	0.64 ± 0.01 Syn	0.67 ± 0.04 Syn	1.29 ± 0.13 Ant	0.92 ± 0.01 Add	0.86 ± 0.07 Syn	1.27 ± 0.13 Ant	1.11 ± 0.10 Ant	0.88 ± 0.07 Syn	0.92 ± 0.07 Add	0.85 ± 0.05 Syn	0.95 ± 0.07 Add	1.00 ± 0.09 Add	0.97 ± 0.07 Add
Mixture of DAS (6.58 nM)		0.54 ± 0.03 Syn	0.88 ± 0.17 Syn	0.85 ± 0.07 Syn	0.83 ± 0.05 Syn	0.79 ± 0.04 Syn	0.79 ± 0.08 Syn	0.82 ± 0.06 Syn	0.92 ± 0.17 Add	0.83 ± 0.07 Syn	0.96 ± 0.04 Add	0.91 ± 0.07 Add	0.90 ± 0.05 Add
Mixture of HT-2 (21.22 nM)			0.87 ± 0.04 Syn	0.83 ± 0.03 Syn	0.80 ± 0.06 Syn	0.93 ± 0.01 Add	0.87 ± 0.05 Syn	0.90 ± 0.05 Add	0.94 ± 0.04 Add	0.90 ± 0.04 Add	0.91 ± 0.04 Add	0.92 ± 0.02 Add	0.92 ± 0.06 Add

Data are expressed as mean values ± SEM of independent experiment (n = 3), each with six technical replicates. Combination index (CI) < 0.9, 0.9 ≤ CI ≤ 1, and CI > 1 indicate synergism (Syn), additivity (Add), and antagonism (Ant), respectively. AME, alternariol-9-methyl ether; AOH, alternariol; BEA, beauvericin; DAS, diacetoxyscirpenol; DON, deoxynivalenol; ENN A, enniatin A; ENN A1, enniatin A1; ENN B, enniatin B; ENN B1, enniatin B1; HT-2, HT-2 toxin; MPA, mycophenolic acid; T-2, T-2 toxin; TEN, tentoxin; ZEA, zearalenone.

**Table 6:** Mechanism of action of combined exposition to mycotoxins and silibinin. Mechanism of action is evaluated by combination indexes of mycotoxins on HEK 293T cells pretreated with silibinin 2 h prior to exposure to T-2, HT-2 and DAS.

Silibinin Concentration (µM)	109.00	54.5	27.3	13.6	6.8	3.4	1.7	0.9
T-2 exposure	1.00 ± 0.08 Add	0.91 ± 0.04 Add	1.41 ± 0.11 Ant	1.82 ± 0.06 Ant	1.46 ± 0.14 Ant	0.99 ± 0.02 Add		
HT-2 exposure	1.08 ± 0.03 Add	1.03 ± 0.04 Add	1.06 ± 0.07 Add	1.25 ± 0.08 Ant	1.57 ± 0.15 Ant	1.27 ± 0.11 Ant	1.10 ± 0.02 Add	
DAS exposure	1.14 ± 0.01 Ant	1.15 ± 0.02 Ant	1.48 ± 0.08 Ant	1.56 ± 0.13 Ant	1.50 ± 0.04 Ant	1.21 ± 0.05 Ant	1.23 ± 0.06 Ant	1.00 ± 0.04 Add

Data are expressed as mean values ± SEM of independent experiment (n = 3), each with six technical replicates. Combination index (CI) < 0.9, 0.9 ≤ CI ≤ 1, and CI > 1 indicate synergism (Syn), additivity (Add), and antagonism (Ant), respectively. DAS, diacetoxyscirpenol; HT-2, HT-2 toxin; T-2, T-2 toxin.

**Table 7:** Transepithelial transport of mycotoxins through Caco-2 cells (%)

Mycotoxins	Apical Medium	Caco-2 Cells	Basolateral Medium	Raw 264.7 Cells	Total
AME	35.92 ± 1.53^c^	0.49 ± 0.02^ab^	17.68 ± 0.86^bcd^	0.01 ± 0.00^ab^	53.94 ± 1.59^bc^
AOH	39.00 ± 22.02^cd^	0.04 ± 0.02^a^	59.97 ± 2.86^h^	0.07 ± 0.02^h^	99.08 ± 24.47^e^
BEA	10.40 ± 3.89^a^	10.36 ± 0.98^d^	2.31 ± 0.24^a^	0.57 ± 0.03^c^	23.65 ± 3.33^a^
DAS	29.61 ± 0.87^abc^	0.02 ± 0.00^a^	40.12 ± 0.50^f^	0.02 ± 0.00^a^	69.77 ± 0.99^cd^
DON	73.33 ± 2.90^f^	0.00 ± 0.00^a^	21.48 ± 0.91^cde^	0.01 ± 0.00^a^	94.83 ± 2.14^e^
ENN-A	15.08 ± 5.18^ab^	10.76 ± 0.43^d^	16.18 ± 3.17^b^	0.29 ± 0.04^b^	42.31 ± 5.00^a^
ENN-A1	9.52 ± 3.02^a^	2.09 ± 0.28^c^	13.06 ± 0.45^b^	0.22 ± 0.03^b^	24.89 ± 3.24^a^
ENN-B	24.08 ± 3.20^abc^	1.15 ± 0.25^b^	25.49 ± 4.31^e^	0.31 ± 0.03^b^	51.02 ± 7.70^bc^
ENN-B1	10.46 ± 2.43^a^	0.51 ± 0.03^ab^	28.16 ± 3.37^e^	0.60 ± 0.10^c^	39.72 ± 4.74^ab^
HT-2	72.63±2.25^f^	0.01 ± 0.00^a^	49.18 ± 1.80^g^	0.01 ± 0.00^a^	121.83 ± 1.33^f^
MPA	57.27 ± 0.79^ef^	0.00 ± 0.00^a^	39.77 ± 0.52^f^	0.02 ± 0.00^a^	97.07 ± 0.27^e^
T-2	11.63 ± 0.62^a^	0.01 ± 0.00^a^	28.13 ± 0.48^e^	0.01 ± 0.00^a^	39.77 ± 0.98^b^
TEN	28.07 ± 0.62^abc^	0.00 ± 0.00^a^	7.25 ± 0.72^ab^	0.00 ± 0.00^a^	35.33 ± 0.10^ab^
ZEA	32.23 ± 1.71^bc^	0.19 ± 0.01^ab^	54.24 ± 2.38^gh^	0.05 ± 0.01^a^	86.70 ± 0.67^de^
Silibinin	44.34 ± 1.96^cd^	0.00 ± 0.00^a^	23.24 ± 1.79^de^	0.00 ± 0.00^a^	67.58 ± 2.95^cd^

Values are expressed as the mean ± SEM (n = 3). The different letters (e.g., a and b) indicate the significant differences between the mycotoxins in one type of medium/cell based on post-hoc Duncan´s test (*p* ≤ 0.05). Similar or sharing letters (e.g., a and ab) show no significant differences between the mycotoxins in one type of medium/cell (*p* ≤ 0.05). AME, alternariol-9-methyl ether; AOH, alternariol; BEA, beauvericin; DAS, diacetoxyscirpenol; DON, deoxynivalenol; ENN A, enniatin A; ENN A1, enniatin A1; ENN B, enniatin B; ENN B1, enniatin B1; HT-2, HT-2 toxin; MPA, mycophenolic acid; T-2, T-2 toxin; TEN, tentoxin; ZEA, zearalenone.

## Abbreviations

ABCB1	ATP-binding cassette sub family member 1
AFB1	aflatoxin B1
AME	alternariol-9-methyl ether
AOH	alternariol
ASBT	intestinal bile acid transporter
BEA	beauvericin
BF-2	a fibroblast cell line originally established from the caudal fin of *Lepomis macrochirus* (Bluegill)
C-28/I2	immortalized human chondrocyte
Caco-2	Caucasian colon adenocarcinoma
CI	combination index
CHO-K1	Chinese hamster ovary
DAS	diacetoxyscirpenol
DMEM	Dulbecco’s Modified Eagle’s Medium
DON	deoxynivalenol
EMEM	Minimum Essential Medium
ENN A	enniatin A
ENN A1	enniatin A1
ENN B	enniatin B
ENN B1	enniatin B1
ENNs	enniatins
FB1	fumonisin B1
FBS	fetal bovine serum
HEK-293T	human embryonic kidney 293
HepG2	hepatocellular carcinoma epithelial
HT-2	HT-2 toxin
HT29	human Caucasian colon adenocarcinoma
IC_50_	the mycotoxin concentration that halved the cellular viability
LMP	low melting point
LogP	logarithmic values of octanol–water partition coefficient
LOQ	limit of quantitation
MCF7	human Caucasian breast adenocarcinoma
MDA-MD-231	human breast adenocarcinoma
MDCK	Madin–Darby canine kidney
MDR1	multidrug resistance protein 1
MPA	mycophenolic acid
NADH	1,4-Dihydronicotinamide adenine dinucleotide
OTA	ochratoxin A
PBS	phosphate buffer saline
PepT1	intestinal peptide transporter 1
P-gp	P-glycoprotein
RAW	mouse macrophage
ROS	reactive oxygen species
RSD	relative standard deviation
SF-9	the lepidopteran (*S. frugiperda*) cells obtained from the envelope of pupal ovaries
T-2	T-2 toxin
TEN	tentoxin
TNF-α	tumor necrosis factor alpha
V79	lung fibroblasts from male Chinese hamster
Vero	mammalian kidney epithelial
ZEA	zearalenone
